# Interleukin-34 Regulates Th1 and Th17 Cytokine Production by Activating Multiple Signaling Pathways through CSF-1R in Chicken Cell Lines

**DOI:** 10.3390/ijms19061665

**Published:** 2018-06-05

**Authors:** Anh Duc Truong, Yeojin Hong, Janggeun Lee, Kyungbaek Lee, Dong Yong Kil, Hyun S. Lillehoj, Yeong Ho Hong

**Affiliations:** 1 Department of Animal Science and Technology, Chung-Ang University, Anseong 17546, Korea; truonganhduc84@gmail.com (A.D.T.); lovejin5873@naver.com (Y.H.); dwr91@naver.com (J.L.); elkb1226@gmail.com (K.L.); dongyong@cau.ac.kr (D.Y.K.); 2 Department of Biochemistry and Immunology, National Institute of Veterinary Research, 86 Truong Chinh, Dong Da, Hanoi 100000, Vietnam; 3 Animal Biosciences and Biotechnology Laboratory, Agricultural Research Services, United States Department of Agriculture, Beltsville, MD 20705, USA; hyun.lillehoj@ars.usda.gov

**Keywords:** cytokines, chicken, interleukin-34, CSF-1R, signaling pathway

## Abstract

Interleukin-34 (IL-34) is a newly recognized cytokine with functions similar to macrophage colony-stimulating factor 1. It is expressed in macrophages and fibroblasts, where it induces cytokine production; however, the mechanism of chicken IL-34 (chIL-34) signaling has not been identified to date. The aim of this study was to analyze the signal transduction pathways and specific biological functions associated with chIL-34 in chicken macrophage (HD11) and fibroblast (OU2) cell lines. We found that IL-34 is a functional ligand for the colony-stimulating factor receptor (CSF-1R) in chicken cell lines. Treatment with chIL-34 increased the expression of Th1 and Th17 cytokines through phosphorylation of tyrosine and serine residues in Janus kinase (JAK) 2, tyrosine kinase 2 (TYK2), signal transducer and activator of transcription (STAT) 1, STAT3, and Src homology 2-containing tyrosine phosphatase 2 (SHP-2), which also led to phosphorylation of NF-κB1, p-mitogen-activated protein kinase kinase kinase 7 (TAK1), MyD88, suppressor of cytokine signaling 1 (SOCS1), and extracellular signal-regulated kinase 1 and 2 (ERK1/2). Taken together, these results suggest that chIL-34 functions by binding to CSF-1R and activating the JAK/STAT, nuclear factor κ B (NF-κB), and mitogen-activated protein kinase signaling pathways; these signaling events regulate cytokine expression and suggest roles for chIL-34 in innate and adaptive immunity.

## 1. Introduction

In mammals, interleukin (IL)-34 is expressed in several cell types, such as macrophages, endothelial cells, fibroblasts, neurons, hepatocytes, regulatory T cells, and epithelial cells [1,2,3,4]; it is constitutively expressed in several adult tissues, such as the heart, brain, liver, spleen, thymus, testes, ovaries, prostate, and small intestine [5,6]. Previous reports demonstrated that, although IL-34 has no distinct sequence homology with macrophage colony-stimulating factor (M-CSF), its biological activity is mediated by interaction with the homodimeric M-CSF receptor that is mainly expressed on the cell surface of macrophages [4,7]. Moreover, IL-34 also interacts with receptor-type protein-tyrosine phosphatase ζ, which is primarily expressed on neural progenitors and glial cells but not in fibroblasts [8,9,10]. On the other hand, the biological effects of CSF-1 or IL-34 are mediated by signaling through a single colony-stimulating factor receptor (CSF-1R), which can lead to distinct functional outcomes in mammals. M-CSF plays a key role in the development of cells, such as monocytes, macrophages, dendritic cells, microglia, Langerhans cells, and regulators of bone homeostasis, including hemopoietic and hematopoietic stem cells; IL-34 has an essential role in the development of Langerhans cells, osteoclasts, and microglia, but not monocytes, macrophages, or dendritic cells [4,11,12]. To date, the function of IL-34 has been mainly described in monocytes, macrophages, dendritic cells, Langerhans cells, microglia, and fibroblasts in the contexts of human diseases, cell development, cell differentiation, or cell cycle [3,4,13,14]. Those studies indicated that IL-34 is involved in the maintenance and development of monocytes, macrophages, dendritic cells, and Langerhans cells [15]. In addition, it has been reported as a genetic marker for several human diseases, such as rheumatoid arthritis [16], obesity, chronic inflammation, insulin resistance [6], hepatitis C viral infection [17], influenza A viral infection [18], non-alcoholic fatty liver disease [19], and metastases of hepatocellular carcinoma [20,21]. In mammals, IL-34 induces and regulates IL-1β, IL-6, IL-8, IL-17, IL-23, CD14, and CD68 expression by activating the Janus kinase/signal transducer and activator of transcription (JAK/STAT), PI3K/AKT, JNK1/2, and extracellular signal-regulated kinase 1 and 2 (ERK1/2) signaling pathways [2,14,18]; it regulates pro-inflammatory cytokines and chemokines in several cell types [1,2,3,4].

Chicken IL-34 (chIL-34) was first identified using a bioinformatics approach [22]. Comparative sequence and co-evolution analyses across all vertebrates suggested that chIL-34 and CSF-1 interact with distinct regions of the CSF-1 receptor (CSF-1R) [4,22] and share no overt sequence homology [13], which accounts for the differences in their functions. However, the mechanism of chIL-34 signaling has not been identified to date. In this study, we investigated the expression of IL-34 and its interaction with CSF-1R in chicken cell lines. We also explored how IL-34 is involved in the JAK/STAT, NF-κB, and mitogen-activated protein kinase (MAPK) signaling pathways to modulate cytokine production.

## 2. Results

### 2.1. Effects of chIL-34 on Cell Proliferation

In pilot experiments with a range of concentrations of recombinant chIL-34 (10, 50, 100, 200, 400, and 800 ng/mL), we demonstrated that recombinant chIL-34 (200 ng/mL) had a marked effect on the activation of certain kinases in chicken cell lines. To investigate the effects of recombinant chIL-34 on the proliferation of the macrophage (HD11) and fibroblast (OU2) cell lines, we treated the cells with chIL-34 (200 ng/mL), anti-CSF-1R antibody (10 µg/mL), or a combination of anti-CSF-1R antibody (10 µg/mL) and chIL-34 (200 ng/mL) for 72 h. The number of HD11 and OU2 cells was significantly increased after treatment with chIL-34 compared to the numbers of control, anti-CSF-1R-, or combination-treated cells (Figure S1C). These results indicated that chIL-34 enhanced the proliferation of HD11 and OU2 cells. In addition, we measured the nitric oxide (NO) production induced by chIL-34 (200 ng/mL), anti-CSF-1R antibody (10 µg/mL), or combination anti-CSF-1R antibody and chIL-34 treatment for 72 h. ChIL-34 treatment induced NO production in the HD11 (43.37 µM) and OU2 (40.08 µM) cell lines (Figure S1D). The concentration of NO was higher in HD11 than in OU2 cells after treatment with chIL-34 and higher after treatment with chIL-34 than in the control, anti-CSF-1R, or combined anti-CSF-1R antibody and chIL-34 stimulation conditions. These results suggest that chIL-34 affects cell proliferation and induces the production of reactive oxygen species in the form of NO.

### 2.2. ChIL-34 Regulates CSF-1R Signaling in Chicken Cell Lines

**Figure 1:**
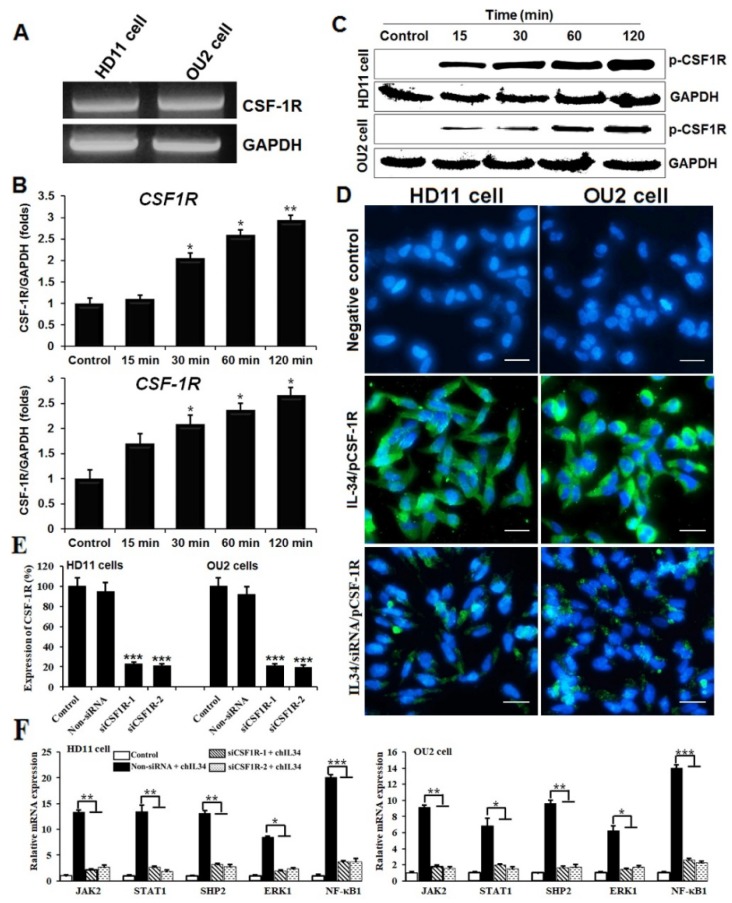
Colony-stimulating factor receptor (CSF-1R) is expressed in chicken cell lines. (**A**) Expression of *CSF-1R* analyzed by real-time quantitative polymerase chain reaction (qRT-PCR) on mRNA derived from chicken cell lines as indicated. (**B**) *CSF-1R* mRNA expression in macrophage (HD11) cells (above) and fibroblast (OU2) cells (below) induced by interleukin-34 (IL-34), measured by qRT-PCR and normalized to glyceraldehyde-3-phosphate dehydrogenase (*GAPDH*). (**C**,**D**) CSF-1R protein expression as demonstrated by western blot analysis and immunocytochemical staining with the CSF-1R antibody in HD11 (left) and OU2 (right) cells using an Alexa Fluor^®^ 488-conjugated goat anti-rabbit IgG (H + L) secondary antibody and 4′,6-diamidino-2-phenylindole (DAPI) (blue). Scale bar represents 10 μm. (**E**) Inhibition of chicken *CSF-1R* mRNA expression by CSF1R-specific siRNA in HD11 and OU2 cell lines. Cells were transfected with non-siRNA, siCSF1R-1, and siCSF-1R-2 for 48 h and subjected to qRT-PCR analysis. (**F**) After transfection with siCSF-1R-1, siCSF1R-2, or non-siRNA, cells were stimulated with recombinant chIL-34 (200 ng/mL) for 24 h (non-siRNA used as a negative control). Data are presented as the mean ± SEM (*n* = 3) of 3 independent experiments. * *p* < 0.05, ** *p* < 0.01, and *** *p* < 0.001.

To study the ligand–receptor system, we first examined if *CSF-1R*, a receptor for IL-34, is expressed in chicken cell lines. We performed real-time quantitative (qRT) polymerase chain reaction (PCR) for *CSF-1R* mRNA in the chicken cell lines HD11 and OU2 and demonstrated that *CSF-1R* was expressed in both cell lines (Figure 1A), whereas *IL-34* was not. The expression of *CSF-1R* in HD11 and OU2 cells as assessed by qRT-PCR was significantly increased after 30 min of treatment with recombinant chIL-34 (Figure 1B). The protein expression level of CSF-1R was confirmed by western blotting (Figure 1C) and by immunofluorescence staining (Figure 1D) using specific antibodies against phosphorylated (p)-CSF-1R. We observed CSF-1R expression and cellular localization in both immune cell types by qRT-PCR, western blot, and immunocytochemical staining after treatment with chIL-34.

Moreover, two small interfering RNA (siRNA) sequences that target CSF-1R intracellular (siCSF-1R-1) and extracellular (siCSF-1R-2) regions were evaluated for their capacity to inhibit expression of chicken *CSF-1R* transcript in HD11 and OU2 cell lines by qRT-PCR after 48 h of transfection (Figure 1E). The siRNA sequences significantly inhibited the expression of *CSF-1R* mRNA in HD11 and OU2 cells, compared to the nonsense siRNA (non-siRNA); non-treated cells were used as a negative control. Inhibition was most efficient with siCSF-1R-1, which inhibited *CSF-1R* mRNA expression by up to 78.62% and 80.67% in HD11 and OU2 cell lines, respectively (Figure 1E). To determine the inhibitory effect of the siCSF-1R-1 and siCSF-1R-2 sequences on the signaling molecules, we transfected the cell lines with non-siRNA, siCSF-1R-1, or siCSF-1R-2 for 48 h and stimulated them with recombinant chIL-34 for 24 h. Both cells transfected with siCSF-1R-1 and siCSF-1R-2 and stimulated with chIL-34 had lower expression levels of *JAK2*, Src homology 2-containing tyrosine phosphatase 2 (*SHP-2*), *STAT1*, *NFKB1*, and *ERK1* mRNA than the cells treated with non-siRNA. In particular, the expression levels of *JAK2*, *SHP-2*, *STAT1*, *NFKB1*, and *ERK1* mRNA were decreased by 84.02%, 86.65%, 78.51%, 77.42%, and 81.51%, respectively, in HD11 cells (Figure 1F, right) and 82.96%, 77.95%, 82.75%, 76.98%, and 83.95% in OU2 cells, respectively, compared to the levels in the cells treated with non-siRNA (Figure 1F, left). In addition, the low expression of CSF-1R protein was confirmed by immunofluorescence staining using specific antibodies against p-CSF-1R after transfection with siCSF-1R-1 and stimulation with recombinant chIL-34 (Figure 1D). Taken together, our results provide new insights into the signaling mechanisms of chIL-34 through CSF-1R.

### 2.3. ChIL-34 Induces the Phosphorylation of STAT1 and STAT3

**Figure 2:**
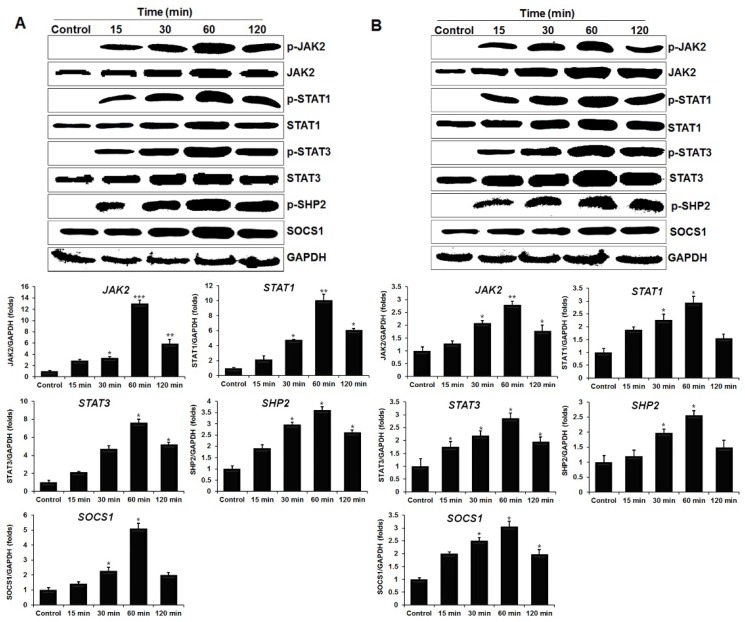
IL-34 activates the Janus kinase/signal transducer and activator of transcription (JAK/STAT) signaling pathway. The HD11 (**A**) and OU2 (**B**) cell lines were stimulated with or without IL-34 for 15, 30, 60, and 120 min. Total lysates were analyzed by western blot with antibodies against total or p-JAK/STAT signaling proteins (top). The changes in mRNA levels of *JAK/STAT* in cells treated with or without IL-34 were measured by qRT-PCR and levels were normalized to those of *GAPDH* (below). Data are presented as the mean ± SEM (*n* = 3) of 3 independent experiments: * *p* < 0.05, ** *p* < 0.01, and *** *p* < 0.001.

Previous reports suggested that IL-34 activates STAT3 phosphorylation in a human fibroblast cell line [23], but the activation of STAT1 phosphorylation by IL-34 has not yet been investigated. To examine the motifs that are phosphorylated in the STATs, we stimulated the chicken cell lines HD11 and OU2 with chIL-34 for various periods. We found that chIL-34 induced serine phosphorylation of STAT1 (Ser^727^) and STAT3 (Ser^727^) within 15 min in both cell lines (Figure 2). The levels of p-STAT1 (Ser^727^) and -STAT3 (Ser^727^) reached their maxima at 60 min, which was consistent with the qRT-PCR results (Figure 2A,B). In particular, the expression of *STAT1* and *STAT3* mRNA was upregulated by 10.1- and 7.5-fold, respectively, in HD11 cells, and 2.9- and 2.8-fold, respectively, in OU2 cells 60 min after treatment with chIL-34 (Figure 2). Moreover, strong cellular localization of p-STAT1/3 proteins was observed by immunocytochemical staining in each immune cell type after chIL-34 treatment (Figure S2). The p-STAT1 and p-STAT3 proteins (green) were more strongly expressed in the cytoplasm of the chIL-34-treated cell lines than in that of the untreated cells, with a particularly strong induction in the HD11 cell line. Taken together, these data indicated that p-STAT1 (Ser^727^) and p-STAT3 (Ser^727^) are involved in the intracellular signaling induced by chIL-34 in both cell lines tested.

### 2.4. IL-34 Activates the Phosphorylation of JAK2, SHP-2, and Suppressor of Cytokine Signaling 1 (SOCS1) in Chicken Cell Lines

JAKs are known to be responsible for STAT phosphorylation in response to cytokine stimulation [24]; we investigated the effect of chIL-34 on JAK2 phosphorylation (Tyr^1007^/Tyr^1008^) in the chicken cell lines. We treated the cells with or without chIL-34, then performed western blots with anti-JAK2 and anti-p-JAK2 (Tyr^1007^/Tyr^1008^) antibodies, as shown in Figure 2. Compared with the unstimulated controls, the cells that received recombinant chIL-34 (200 ng/mL) had a strong induction of JAK2 tyrosine phosphorylation after 15 min, which peaked at 60 min and remained until 120 min, in both cell lines (Figure 2). To further assess the functional role of JAK2 in chIL-34 signaling, we performed qRT-PCR and immunocytochemical staining (Figure 2 and Figure S2). We found that p-JAK2 (Tyr^1007^/Tyr^1008^) expression (green) was strongly increased in the cytoplasm of both cell lines 60 min after treatment with chIL-34, compared to expression in the untreated control cells, and that *JAK2* mRNA was markedly increased after 30 min and reached the highest levels after 60 min; there was a greater increase in the HD11 cell line (Figure 2 and Figure S2). These results indicate that chIL-34 regulates the expression and subcellular localization of p-JAK2 (Tyr^1007^/Tyr^1008^), resulting in the activation of these signaling molecules and their associated pathways.

SHP-2, a cytoplasmic SH2 domain-containing protein tyrosine phosphatase, is involved in the signaling pathways of a variety of growth factors and cytokines [25]. To assess the levels of p-SHP-2 (Tyr^542^) in the chicken cell lines, we measured p-SHP-2 (Tyr^542^) after treatment with chIL-34 by western blot, qRT-PCR, and immunocytochemical staining (Figure 2). The results demonstrated that chIL-34 induced p-SHP-2 (Tyr^542^), as shown in Figure 2. We also detected substantial levels of suppressor of cytokine signaling 1 (SOCS1) protein after 60 min, as with STAT1, STAT3, JAK2, and SHP-2. The expression of *SOCS1* mRNA was significantly upregulated by 5.0- and 3.0-fold in HD11 and OU2 cells, respectively, after chIL-34 treatment (Figure 2). These results demonstrated that chIL-34 induced and activated the JAK/STAT signaling pathway, which may activate downstream cytokine production, in chicken HD11 and OU2 cells.

### 2.5. Phosphorylation of ERK1/2 and p-Mitogen-Activated Protein Kinase Kinase Kinase 7 (TAK1) by chIL-34

**Figure 3:**
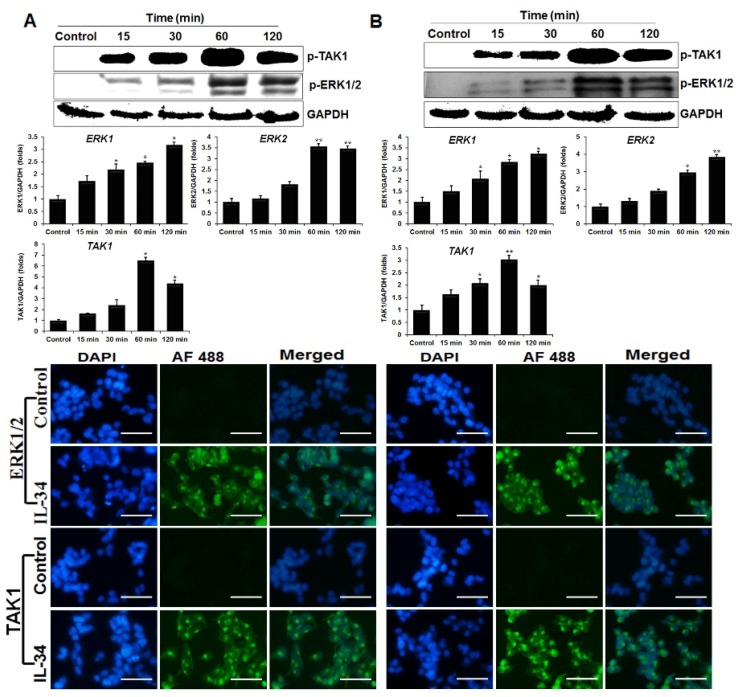
IL-34 induces phosphorylation of extracellular signal-regulated kinase 1 and 2 (ERK1/2) and p-mitogen-activated protein kinase kinase kinase 7 (TAK1). The HD11 (**A**) and OU2 (**B**) cell lines were treated with or without IL-34 for 15, 30, 60, and 120 min. Total lysates were analyzed by western blot with antibodies against p-ERK1/2 and p-TAK1 (top). The changes in mRNA levels for ERK1/2 and TAK1 in cells treated with or without IL-34 were analyzed by qRT-PCR in chicken cell lines and levels were normalized to those of GAPDH (middle). Data are presented as the mean ± SEM (*n* = 3) of 3 independent experiments: * *p* < 0.05 and ** *p* < 0.01. Immunocytochemical analysis of p-ERK1/2 and p-TAK1 signaling proteins in HD11 (left) and OU2 (right) cells. Untreated cells and those treated with chIL-34 were incubated with primary antibody, Alexa Fluor*^®^* 488-conjugated goat anti-rabbit IgG (H + L) secondary antibody, and DAPI (blue). Scale bar represents 25 μm.

The ERK1/2 MAPK signaling pathway regulates cell proliferation, differentiation, and transformation [26]. Previous reports demonstrated that human IL-34 regulated the ERK1/2 MAPK signaling pathway and induced cytokine production [27,28,29]; therefore, we investigated whether chIL-34 affected two major MAPK pathway-related genes, p-ERK1/2 (Thr^202^/Tyr^204^), and p-mitogen-activated protein kinase kinase kinase 7 (TAK1) (Ser^933^) in the chicken cell lines using western blotting, qRT-PCR, and immunocytochemical staining (Figure 3). Compared with the unstimulated controls, the chIL-34-treated (200 ng/mL) cell lines had higher levels of p-ERK1/2 after 15 min, which reached maximum levels by 120 min (Figure 3). Similarly, chIL-34 strongly induced p-TAK1 (Ser^933^), with maximal induction after 60 min (Figure 3). We found by qRT-PCR and immunocytochemical staining that p-ERK1/2 (Thr^202^/Tyr^204^) and TAK1 (Ser^933^) proteins (visualized as green fluorescence) were strongly upregulated in the cytoplasm after treatment with chIL-34 for 60 min, as compared to controls, and that mRNA expression was highly increased after 60 min, particularly in the HD11 cell line (Figure 3). These results indicate that chIL-34 increases the expression and alters the subcellular localization of p-ERK1/2 (Thr^202^/Tyr^204^) and TAK1 (Ser^933^).

### 2.6. ChIL-34 Activates NF-κB1 and MyD88

**Figure 4:**
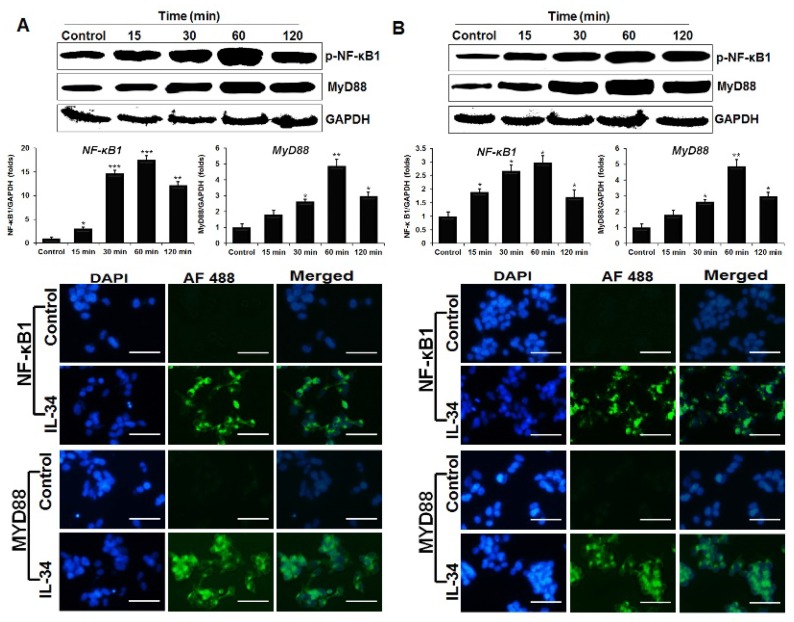
IL-34 induces phosphorylation of NF-κB1 and MyD88. The HD11 (**A**) and OU2 (**B**) cell lines were treated with or without IL-34 for 15, 30, 60, and 120 min. Total lysates were analyzed by western blot with antibodies against p-NF-κB1 and MyD88 (top). The changes in the levels of mRNA for *NFKB1* and *MYD88* in cells treated with or without chIL-34 were measured by qRT-PCR and levels were normalized to those of *GAPDH* (middle). Data are presented as the mean ± SEM (*n* = 3) of 3 independent experiments: * *p* < 0.05, ** *p* < 0.01, and *** *p* < 0.001. Immunocytochemical analysis of p-NF-κB1 and MyD88 signaling proteins in HD11 (left) and OU2 (right) cells. Both untreated cells and those treated with chIL-34 were incubated with primary antibody, Alexa Fluor^®^ 488-conjugated goat anti-rabbit IgG (H + L) secondary antibody and DAPI (blue). Scale bar represents 25 μm.

NF-κB is a protein complex that plays an important role in the regulation of transcription, cytokine production, cell survival, cellular responses, and immune responses to infection [30]. To explore if NF-κB signaling pathways are activated in chicken HD11 and OU2 cell lines treated with recombinant chIL-34, we evaluated p-NF-κB1 (Ser^192^) and MyD88 expression by western blot and immunocytochemical staining with phospho-specific antibodies and qRT-PCR. The p-NF-κB1 (Ser^192^) and MyD88 antibodies displayed a similar staining pattern in the cells after treatment with chIL-34 (Figure 4). NF-κB1 (Ser^192^) and MyD88 expression reached maximum levels after 60 min of treatment, as indicated by western blotting, in both cell lines. These genes were significantly induced by 17.45- and 2.96-fold (*NFKB1*), and 5.45- and 4.84-fold (*MYD88*) in the HD11 and OU2 cell lines, respectively (Figure 4). Moreover, the expression levels of p-NF-κB1 (Ser^192^) and MyD88 protein (green) were strongly increased by chIL-34 in the nucleus or cytoplasm compared with the expression in untreated cells. These results suggested that chIL-34 regulates the expression and subcellular localization of p-NF-κB1 (Ser^192^) and MyD88 and indicated that chIL-34 activates NF-κB1 and MyD88 signaling in chicken cell lines.

### 2.7. Treatment with chIL-34 Upregulates Cytokine Expression

**Figure 5:**
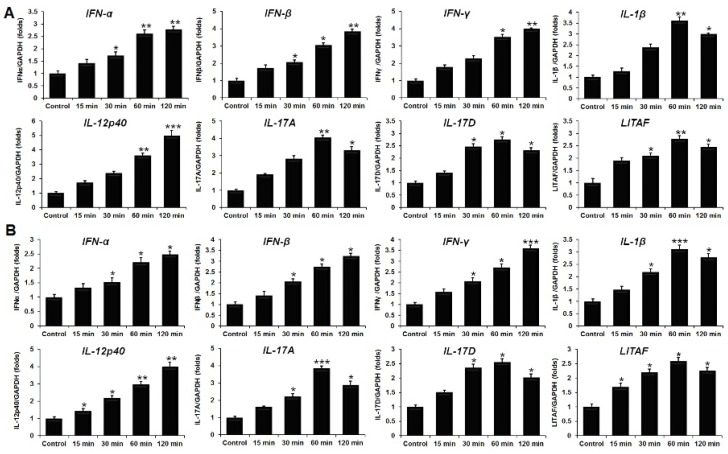
The expression levels of cytokines induced by chicken IL-34 (chIL-34) in HD11 (**A**) and OU2 (**B**) cell lines. Levels are normalized to those of GAPDH. Data are presented as the mean ± SEM (*n* = 3) of 3 independent experiments: * *p* < 0.05, ** *p* < 0.01, and *** *p* < 0.001.

To determine cytokine gene expression in HD11 and OU2 cell lines, qRT-PCR was performed following recombinant chIL-34 treatment. The expression levels of cytokine genes in the HD11 and OU2 cell lines with and without treatment are shown in Figure 5. When the HD11 macrophage cell line was co-cultured with chIL-34 protein, the expression levels of the Th1-type cytokines *IFN-α*, *IFN-β*, *IFN-γ*, and *IL-1β* were significantly increased, particularly after 60 or 120 min of treatment (Figure 5A). We also examined the effects of chIL-34 on the pro-inflammatory mediator lipopolysaccharide-induced tumor necrosis factor (TNF) factor (*LITAF*); *LITAF* was upregulated by 2.74-fold in the HD11 cell line after treatment with chIL-34 for 60 min (Figure 5A). Moreover, Th17-type cytokine genes, including *IL-12p40*, *IL-17A*, and *IL-17D*, were highly upregulated in the HD11 cell line by 4.97-, 4.0-, and 2.73-fold, respectively, following chIL-34 treatment (Figure 5A). In addition, we investigated the expression of Th1 and Th17 cytokines in the OU2 cell line following treatment with chIL-34 protein. The results were similar to those in the HD11 cell line (Figure 5B); however, gene expression in HD11 cells was generally higher than in OU2 cells (Figure 5).

Moreover, we measured the Th1 (IFN-γ) and Th17 (IL-17A and IL-12p40) cytokine production in the supernatants of the chIL-34-treated cultures by enzyme-linked immunosorbent assays (ELISA) (Table 1). The results revealed significantly higher IFN-γ protein levels (415.09 and 164.36 ng/mL in HD11 and OU2 cell lines, respectively) in IL-34-treated cells compared to mock controls (17–22 ng/mL) (Table 1). Moreover, Th17 cytokine protein production was significantly upregulated by chIL-34 treatment in all tested cells (Table 1). In particular, IL-17A and IL-12p40 were produced at markedly high levels in the HD11 cell line (259.28 and 247.66 ng/mL, respectively) and the OU2 cell line (148.39 and 106.73 ng/mL, respectively) (Table 1). These results indicated that chIL-34 more strongly induced Th1 (IFN-γ) and Th17 (IL-17A and IL-12p40) cytokines in HD11 cells than in OU2 cells. Taken together, these results suggest that chIL-34 induces Th1 and Th17 cytokine production in vitro in the HD11 and OU2 cell lines.

### 2.8. Blockade of CSF-1R Reduces Cytokine Production in Chicken Cell Lines

To confirm the role of IL-34 in the regulation of cytokine production through CSF-1R signaling, we cultured both chicken cell lines with a neutralizing CSF-1R antibody and measured Th1 (IFN-γ) and Th17 (IL-17A and IL-12p40) cytokine expression by ELISA. In each experiment, treatment with the CSF-1R antibody reduced Th1 (IFN-γ) and Th17 (IL-17A and IL-12p40) cytokine secretion by 60–83% (Table 1). These results indicated that chIL-34 activates and induces cytokine production in chicken cell lines through CSF-1R signaling. The differing levels of expression by the HD11 and OU2 cell lines may reflect cell-specific differences in responsiveness to stimulation or the capacity to produce cytokines.

## 3. Discussion

**Figure 6:**
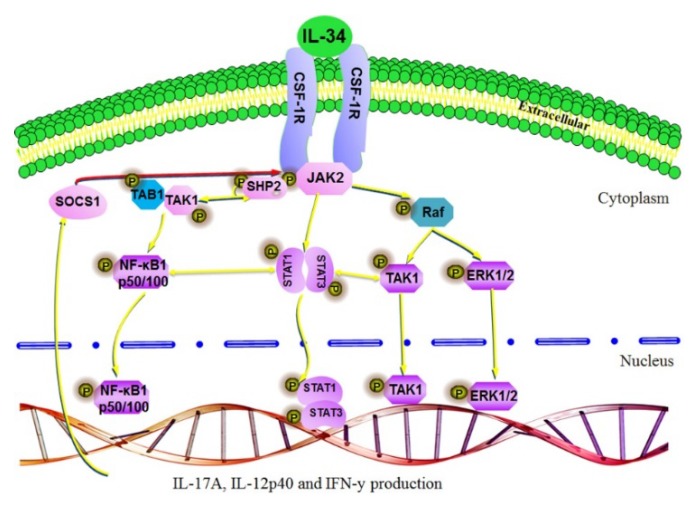
Summary and model for the potential function of the chIL-34 signaling pathway.

In this study, we analyzed the signaling pathways activated by chIL-34 in the chicken cell lines HD11 and OU2, as summarized in Figure 6, and compared the functions of chicken and mammalian IL-34 (Table 2). Our results indicated that chIL-34 binds to CSF-1R; this association, which induces the phosphorylation of JAK2 (Tyr^1007^/Tyr^1008^), is required for chIL-34 signaling. After phosphorylation by JAKs, STAT proteins form homo- and heterodimeric complexes and translocate into the nucleus, where they bind to specific consensus sequences of target gene promoters and modulate gene expression [31,32,33]. We also detected phosphorylation of p-STAT1 (Ser^727^) and p-STAT3 (Ser^727^) by western blotting and immunocytochemical staining and STAT1/3 mRNA expression by qRT-PCR (Figure 2 and Figure S2). These data indicate that chIL-34 activated JAK/STAT proteins through CSF-1R signaling in chicken cell lines. Moreover, the SH2 domain of SHP-2 is known to bind to JAK2 [25], and STAT1 and STAT3 are recruited by its SH2 domain to a phosphotyrosine site in CSF-1R [34]. The expression of p-SHP-2 (Tyr^542^) protein and mRNA showed a pattern similar to p-JAK2 (Tyr^1007^/Tyr^1008^) and p-STAT1/3 (Ser^727^) after chIL-34 treatment in both cell lines. These results show that chIL-34 binds to CSF-1R and activates p-JAK2 (Tyr^1007^/Tyr^1008^), p-STAT1/3 (Ser^727^), and p-SHP-2 (Tyr^542^).

SOCS1 plays an important role in the negative regulation of JAK/STAT signaling [24]. The *SOCS1* gene is activated in response to STAT-mediated mechanisms and the protein interacts with the catalytic domains of JAK proteins [24]. In the present study, upregulated SOCS1 mRNA and protein were observed in HD11 and OU2 cell lines after chIL-34 treatment. These results suggest that SOCS1 regulates the signaling pathway downstream of chIL-34, which is important to produce pro-inflammatory cytokines. Therefore, this study provides the first evidence indicating the possible role of the JAK/STAT pathway in the signaling mechanism of chIL-34 in chicken cell lines.

Moreover, the NF-κB signaling pathways are central regulators of innate and adaptive immune responses in humans [36,37]. IL-34 induces cytokine expression through the activation of the NF-κB signaling pathway [3,38]; however, the role of NF-κB signaling induced by IL-34 in the chicken was previously unknown. Therefore, we demonstrated by western blotting and immunocytochemical staining that chIL-34 activates NF-κB1 and MyD88, which is a key regulator of NF-κB signaling pathways. Previous studies have demonstrated that the interaction between STAT1 and NF-κB1 plays a key role in regulating gene promoters and increases innate and adaptive immune responses, such as the production of chemokines [36] and Th1 and Th17 cytokines [30,39]. In addition, STAT3/NF-κB1 signaling plays an important role the development and progression of colon, gastric, and liver cancers, as well as the control of immune responses [37,40]. Our findings indicated that chIL-34 induced JAK/STAT and NF-κB signaling and interaction and that the communication between STAT1/3 (Ser^727^) and NF-κB1 (Ser^933^) may control the expression of the cytokines and immune mediators shown in Figure 5 and Figure 6. On the other hand, p-TAK1 and p-ERK1/2 MAPK are activated by pro-inflammatory cytokines and the Toll-like receptor family [41,42]. TAK1 works with TGF-β-activated kinase binding protein family genes to activate downstream kinases, leading to NF-κB1 activation and MAPK signaling [41,42]. Previous research indicated that TAK1 induced the serine phosphorylation of STAT3 [41,42], and that the association between TAK1 and JAK led to the regulation of the expression of pro-inflammatory cytokines through NF-κB1 activation [43]. In addition, the associations among the ERK1/2, MAPK (Thr^202^/Tyr^204^), JAK2 (Tyr^1007^/Tyr^1008^) [44], STAT1/3 (Ser^727^) [45,46], SHP-2 (Tyr^542^) [31], PI3K/AKT, and p38 signaling pathways [26] participate in the control of cell differentiation, development, proliferation, and pro-inflammatory cytokine production in several cell types, such as epithelial cells and macrophages. Our results indicated that chIL-34 activates JAK/STAT, NF-κB, and MAPK signaling pathways; the JAK/STAT signaling pathway may play a role in cross-talk between the NF-κB and MAPK signaling pathways for chIL-34-induced cytokine production.

In humans, IL-34 may have a pro-inflammatory effect and contribute to inflammation in several diseases [5]. We found that the expression levels of IFN-γ, IL-17A, and IL-12p40 were more highly increased in the HD11 than the OU2 cell line after treatment with chIL-34, and were more notably reduced by the CSF-1R antibody (Table 2). In addition, chIL-34 induced other Th1 (IFNα, IFNβ, and IL-1β) and Th17 (IL-17D) cytokines and the pro-inflammatory mediator LITAF; these results indicated that chIL-34 may be pro-inflammatory and activate the JAK/STAT, NF-κB, and MAPK signaling pathways, thereby upregulating the expression of Th1 and Th17 cytokines. Furthermore, this study indicates that IL-34 is essential for cell-mediated immune responses and could be a potential therapeutic protein in chickens.

In summary, this is the first report to describe the signal transduction downstream of chIL-34. Our results showed that chIL-34 activates JAK2, tyrosine kinase 2 (TYK2), STAT1/3, NF-κB, and MAPK signaling pathways through CSF-1R signaling, which upregulates the expression of pro-inflammatory cytokines in chicken macrophage and fibroblast cell lines. In conclusion, our findings demonstrate the role of chIL-34 in controlling Th1 and Th17 pro-inflammatory gene transcription via multiple signaling pathways.

## 4. Materials and Methods

### 4.1. ChIL-34 Protein Production

To clone full-length chIL-34, the predicted chIL-34 coding sequence (GenBank accession # XM_003641892) was amplified from total RNA of the intestinal mucosal layer using the restriction enzyme-anchored primers in Table 3. Total RNA was isolated using TRIzol^®^ reagent (Invitrogen, Carlsbad, CA, USA) as described [47] from the intestinal mucosal layer of White Leghorn chickens, kindly provided by the Animal Biosciences and Biotechnology Laboratory (Beltsville, MD, USA) of the USDA Agricultural Research Service; the RNA was stored at −80 °C. The first-strand cDNA was subsequently synthesized using a Maxima First Strand cDNA Synthesis Kit (Thermo Fisher Scientific, Waltham, MA, USA). PCR was performed to amplify the full-length chIL-34 cDNA under the following conditions: Initial denaturation at 94 °C for 3 min; 35 cycles of denaturation at 94 °C for 30 s, annealing at 55 °C for 30 s, and extension at 72 °C for 30 s; and a final extension at 72 °C for 5 min. The methods for cloning, transformation, and protein purification were previously described [47]. Briefly, the recombinant chIL-34 protein was purified using HisPur™ Cobalt Resin (Thermo Fisher Scientific) in the first purification step, per the manufacturer’s instructions. To remove the endotoxin contaminants, we combined affinity chromatography with a non-ionic detergent washing step as previously described [48]. The purified protein was concentrated and the buffer was changed by ultrafiltration using a 3000-molecular weight-cutoff membrane (EMD Millipore, Billerica, MA, USA). The samples were dialyzed in phosphate-buffered saline (PBS; pH 7.2) overnight using SnakeSkin™ dialysis tubing (Thermo Fisher Scientific) with stirring, then analyzed by SDS-PAGE. ELISAs demonstrated that the 2-step purification of the recombinant protein facilitated a drastic depletion of endotoxin contaminants (<0.006 EU/mL) (MyBioSource, San Diego, CA, USA; Figure S1A), performed according to the manufacturer’s instructions. A single chIL-34 protein band with an apparent molecular mass of approximately 41.0 kDa was observed by western blot using the horseradish peroxidase (HRP)-conjugated anti-His (C-Term) antibody (1:3000, Invitrogen, Carlsbad, CA, USA) (Figure S1B), as described below. The larger size than predicted (20.3 kDa) was due to the 3 epitope tags (polyhistidine, S-protein, and thioredoxin) in the recombinant protein.

### 4.2. Chicken Cell Lines and Treatment with chIL-34

The macrophage (HD11) [49] and fibroblast (OU2) [50] cell lines were kindly provided by Dr. Hyun S. Lillehoj’s Laboratory at the Agricultural Research Service, USDA. Cells were cultured in Dulbecco’s modified Eagle’s medium (DMEM; Invitrogen) containing 100 IU/mL penicillin, 100 mg/mL streptomycin, and 10% heat-inactivated fetal bovine serum (FBS, Invitrogen) in a humidified 5%-CO_2_ atmosphere at 41 °C. To elucidate chIL-34 activity, HD11 and OU2 cells (1.0 × 10^6^/well) were stimulated in 6-well plates with recombinant chIL-34 (200 ng/mL) for 15, 30, 60, and 120 min, in DMEM with 10% FBS plus antibiotics, unless otherwise indicated. In all experiments, the control groups were cultured in only DMEM with 10% FBS plus antibiotics. The final concentration of chIL-34 was based on pilot experiments that demonstrated a marked effect of the recombinant protein on the activation of certain kinases. After incubation, the cell supernatants were collected to evaluate cytokine production by ELISA, then cells were washed twice with ice-cold PBS and harvested in ice-cold RIPA buffer (50 mM Tris-HCl, pH 7.5; 150 mM NaCl; 2 mM EDTA; 100 mM NaF; 0.1% SDS; 0.5% sodium deoxycholate; 1% Triton X-100; 10 mM sodium pyrophosphate; and 10 mM sodium orthovanadate) containing complete™ EDTA-free protease inhibitor cocktail (Thermo Fisher Scientific) for protein analysis. Cells were gently lysed for 30 min at 4 °C and centrifuged at 13,000× *g* for 15 min at 4 °C. The protein concentration was determined using the Coomassie (Bradford) Protein Assay Kit (Thermo Fisher Scientific) in microplates, according to the manufacturer’s instructions. Finally, the cells were stimulated with chIL-34 and cultured with or without neutralizing CSF-1R antibody (10 μg/mL) for 72 h in DMEM with 10% FBS plus antibiotics, then cytokine expression levels were evaluated in cell-free culture supernatants by ELISA.

### 4.3. RNA Interference Assay

The chicken cell lines were transfected with 500 pmol chicken CSF-1R-specific small interfering RNA (siCSF-1R-1 and siCSF-1R-2; Bioneer, Daejeon, Korea; Table 1) using Lipofectamine^®^ 3000 transfection reagent (Invitrogen) according to the manufacturer’s instructions and as previously described [51]. Briefly, HD11 and OU2 cells (1 × 10^6^ cells/well) were seeded in 6-well plates and transfected with siRNA–Lipofectamine complexes. After 48 h of transfection, the cells were stimulated with recombinant chIL-34 for another 24 h. Total RNA isolation was performed as described below and RNA was used for the analysis of signaling molecules by qRT-PCR. Non-siRNA was used as a negative control for non-sequence-specific effects (Bioneer).

### 4.4. ELISA

We coated a 96-well plate (Nunc MaxiSorp^®^, Nunc, Wiesbaden, Germany) with dilutions (1:500) of monoclonal anti-IFN-γ, anti-IL-12p40, or anti-IL-17A antibodies for 7 days at 4 °C, as previously described [51]. After blocking with 5% skim milk for 1 h at room temperature (RT, 25 °C), the plate was incubated with culture supernatants or different dilutions of recombinant IFN-γ, IL-12p40, or IL-17A overnight at 4 °C. Following incubation with a biotinylated-IFN-γ, -IL-12p40 or -IL-17A antibody (1:500), HRP-conjugated streptavidin (1:5000; Thermo Fisher Scientific) was added. The substrate 3,3′,5,5′-tetramethylbenzidine (100 µL/well; Thermo Fisher Scientific) was used as a chemiluminescent substrate and luminescence was measured in a Hybrid Microplate Reader (Epoch, BioTek Instruments, Winooski, VT, USA).

### 4.5. Western Blotting

Protein (100 μg) was mixed at a 1:1 *v*/*v* ratio with 2× sample buffer (62.5 mM Tris-HCl, pH 6.8; 2% SDS; 6 M urea; 10% β-mercaptoethanol; and 20% glycerol) and heated to 100 °C for 5 min. Samples were electrophoresed on 12% Tris-glycine SDS-PAGE gels and transferred to polyvinylidene fluoride membranes (GE Healthcare, Marlborough, MA, USA). Membranes were blocked with 5% skim milk (Thermo Fisher Scientific) or 3% bovine serum albumin (BSA; Sigma-Aldrich, St. Louis, MO, USA) in PBS (pH 7.4) containing 0.05% TWEEN^®^ 20 (PBST) for 3 h at RT. Primary antibodies (1:1000; against STAT1/3, JAK2, SOCS1, TYK2, TAK1, SHP-2, CSF-1R, NF-κB1, MyD88, ERK1/2, and glyceraldehyde-3-phosphate dehydrogenase [GAPDH]) were prepared in either 2% skim milk or 0.5% BSA in PBST overnight at 4 °C. Membranes were washed with PBST and treated with the HRP-linked anti-rabbit secondary antibodies (1:4000; Sigma-Aldrich) or goat anti-mouse IgG HRP conjugate (1:4000; Thermo Fisher Scientific) in 2% skim milk or 0.5% BSA in PBST for 2 h at RT. Subsequently, the membranes were developed using western Lightning^®^ Plus-ECL (Thermo Fisher Scientific) on Hyperfilm™ (GE Healthcare).

### 4.6. RNA Extraction and cDNA Synthesis

Cells were washed with ice-cold PBS and total RNA was extracted using TRIzol^®^ (Invitrogen), according to the manufacturer’s instructions. RNA was diluted with 20 µL RNase-free H_2_O and the concentration was determined using the Hybrid Microplate Reader (BioTek Instruments). For cDNA synthesis, up to 2 µg RNA was treated with 1.0 U DNase I and 1.0 μL of 10× reaction buffer (Thermo Fisher Scientific), then incubated for 30 min at 37 °C. Subsequently, to inactivate DNase I, 50 mM EDTA (1.0 μL) was added and the mixture was heated to 65 °C for 10 min, then reverse-transcribed using the Maxima First Strand cDNA Synthesis Kit (Thermo Fisher Scientific), according to the manufacturer’s recommendations.

### 4.7. qRT-PCR Gene Expression Assay

To analyze the expression of cytokines, we designed primers using Lasergene software (DNASTAR, Madison, WI, USA) (Table 1) and performed qRT-PCR using 2× Power SYBR^®^ Green Master Mix (Roche, Indianapolis, IN, USA), according to the manufacturer’s instructions, with the LightCycler^®^ 96 system (Roche). The cycle number when the fluorescence first reached a preset threshold was used to quantify the initial concentration of the individual templates for the expression of the mRNA of genes of interest. Chicken *GAPDH* was used as an internal control gene to normalize for RNA quantity. The relative gene-specific expression was calculated using the 2^−ΔΔ*C*t^ method after normalization to *GAPDH* [52]. All qRT-PCRs were performed in triplicate.

### 4.8. Immunocytochemistry

We performed immunocytochemistry using chamber slides, as previously described [53,54,55]. Briefly, cells (2.0 × 10^3^/well) were cultured in DMEM with 10% FBS plus antibiotics in a chamber slide (Thermo Fisher Scientific), with or without recombinant chIL-34 (200 ng/mL), for 1 h in a humidified 5%-CO_2_ atmosphere incubator at 41 °C. Then, the cells were fixed in 4% paraformaldehyde/PBS (pH 7.4) for 15 min and incubated in PBS containing 0.25% Triton™ X-100 for 10 min at RT. Thereafter, the cells were blocked with blocking buffer (200 µL/well; Thermo Fisher Scientific) for 45 min at RT. Following overnight incubation with the primary antibody (1:500; against STAT1/3, JAK2, SOCS1, TAK1, CSF-1R, NF-κB1, MyD88, or ERK1/2) overnight at 4 °C, cells were incubated with Alexa Fluor^®^ 488-conjugated secondary antibody (2 µg/mL, Invitrogen) for 1 h and stained with 4′,6-diamidino-2-phenylindole (DAPI) for 5 min at RT. Finally, images were captured on an EVOS^®^ FLoid^®^ Cell Imaging Station (Life Technologies, Carlsbad, CA, USA).

### 4.9. Reagents

The rabbit anti-chicken p-STAT1 (Ser^727^), rabbit anti-chicken p-STAT3 (Ser^727^), and rabbit anti-chicken p-JAK2 (Tyr^1007^/Tyr^1008^) antibodies were purchased from Santa Cruz Biotech (Dallas, TX, USA). The following reagents were either purchased from or provided by each respective company: Rabbit antibodies against chicken TYK2, p-TAK1 (Ser^192^), p-SHP-2 (Tyr^542^), p-CSF-1R (Tyr^809^), and p-NF-κB1 (Ser^933^, p50/100) and mouse antibodies against chicken JAK2 (Biorbyt, San Francisco, CA, USA); rabbit antibodies against chicken SOCS1, chicken STAT1, and chicken STAT3 and HRP-linked anti-rabbit secondary antibodies (Sigma-Aldrich); mouse antibodies against chicken GAPDH (Abcam, Cambridge, MA, USA); anti-chicken MyD88 antibody (Novus Biologicals, Littleton, CO, USA); Alexa Fluor^®^ 488-conjugated goat anti-rabbit IgG (H *+* L) secondary and HRP-linked anti-His (C-Term) antibodies (Invitrogen); rabbit antibody against chicken p-44/42 MAPK (ERK1/2) (Thr^202^/Tyr^204^) (Cell Signaling Technology, Danvers, MA, USA); mouse polyclonal anti-chicken IL-12p40 antibody (Kingfisher Biotech, St. Paul, MN, USA); and mouse monoclonal anti-chicken IFN-γ [56] and IL-17A [57] antibodies (kindly provided by Hyun S. Lillehoj, USDA). We also purchased EZ-Link™ Sulfo-NHS-LC-Biotin, HRP-conjugated streptavidin, and goat anti-mouse IgG HRP conjugate (Thermo Fisher Scientific) and DAPI (Invitrogen).

### 4.10. Bioactivity Assays

Cells (1.0 × 10^3^ cells/well) were seeded and cultured in DMEM with 10% FBS plus antibiotics in 96-well plates. After overnight culture, recombinant chIL-34 was added at the specified concentrations for 72 h. To measure the NO content, culture medium (100 µL) was incubated with Griess reagent (100 µL; Sigma-Aldrich) at RT for 10 min. Then, the absorbance was measured at 540 nm using a microplate reader, as previously described [58]. The nitrite content was calculated based on a standard curve constructed with NaNO_2_. Cell proliferation was determined with the Cell Counting Kit-8 assay (Dojindo Molecular Technologies, Kumamoto, Japan), according to the manufacturer’s protocol as previously described [59].

### 4.11. Statistical Analysis

Measurement data are presented as the mean ± standard error of the mean (SEM) of at least 3 replicates. Statistical analysis was performed using IBM SPSS software (SPSS 23.0 for Windows; IBM, Chicago, IL, USA). One-way analysis of variance (ANOVA) tests and Duncan’s multiple comparison method were carried out to detect the differences. A *p*-value < 0.05 was considered to be statistically significant.

## Figures and Tables

**Table 1:** Colony-stimulating factor receptor (CSF-1R) neutralization with a CSF-1R antibody decreased recombinant chicken IL-34 (chIL-34)-induced cytokine production in chicken cell lines.

Cytokines (ng/mL)	Control	IL-34	IL-34 + Anti-CSF-1R	% Reduction	Cell Line
IFN-γ	22.59	415.09 ***	160.01 *	61.45	HD11
IL-17A	6.67	259.28 **	101.39 *	60.90	
IL-12p40	4.17	247.66 *	40.95	83.47	
IFN-γ	17.45	164.36 *	51.43	68.71	OU2
IL-17A	4.02	148.39 *	53.97	63.63	
IL-12p40	3.44	106.73 *	32.18	69.85	

Data are presented as the mean ± SEM (*n* = 3) of 3 independent experiments. * *p* < 0.05, ** *p* < 0.01, and *** *p* < 0.001.

**Table 2:** Comparison the function of interleukin (IL)-34 in mammals and chickens.

Category	Mammalian IL-34; Review in [35]	Chicken IL-34
Protein	241 aa, 39 kDa	178 aa, 20.3 kDa
Proliferation	Osteoclast precursors → Microglia → Macrophages → Endothelial cells → Osteosarcoma cells →	Macrophages → Fibroblast cells →
Differentiation	Human CD14+ monocytes ↗ Murine CD11b+ cells ↗ Macrophages ↗ PBMCs ↗ Memory T cells ↗ FDMCs ↗	
Molecular signaling pathways	↑ p-ERK1/2, p-Src, p-p38, p-Akt, p-STAT3, p-JNK, p-NF-κB1	↑ p-STAT1, p-STAT3, p-JAK2, p-TAK1, p-SHP-2, p-NF-κB1, p-ERK1/2, STAT1, STAT3, JAK2, TYK2, SOCS1, MyD88
Th1 cytokines	↑ IL-1β, IFNγ	↑ IFN-α, IFN-β, IFN-γ, IL-1β
Th17 cytokines	↑ IL-12, IL-17	↑ IL-12, IL-17A, IL-17D
Th2 cytokines	↑ IL-10	
Chemokines	↑ CXCL10, CXCL8, CCL2, CCR2, CCL20, MIG	
Pro-inflammatory cytokines	↑ TNF-α, IL-6,	↑ LITAF
Other cytokines	↑ TGF-β, TRAP, VEGF	
Inflammation	IL-34-macrophages ¥ Colon epithelial cells → LPMCs → Microglia ¥ M1 macrophages ¥ Human whole blood →	
Cell surface marker	↑ CD161, membrane IL-1α, CD163	

PBMCs, peripheral blood mononuclear cells; FDMCs, follicular DC-induced monocytic cells; CCL, CC chemokine ligand; LPMCs, lamina propria mononuclear cells; CXCL, chemokine (C-X-C Motif) Ligand; VEGF, vascular endothelial growth factor. “↑”, up-regulation; “→”, promote; “↗”, induce; “¥“, inhibit.

**Table 3:** Primer sequences for qRT-PCR analyses of gene expression levels.

Gene	F/R	Nucleotide Sequence (5′-3′)	Accession No.
*GAPDH*	F	TGCTGCCCAGAACATCATCC	NM_204305
	R	ACGGCAGGTCAGGTCAACAA	
*IL-34*	F	CG*GAATTC*ATGCACCAGGGCTGCGCGGC	XM_003641892
	R	CC*AAGCTT*AGCGGAGTCCCACCGACAGTG	
*STAT1*	F	TTGTAACTTCGCTATTGGTATTCC	NM_001012914
	R	TTCCGTGATGTGTCTTCCTTC	
*STAT3*	F	AGGGCCAGGTGTGAACTACT	NM_001030931
	R	CCAGCCAGACCCAGAAAG	
*SOCS1*	F	CTACTGGGGACCGCTGACC	NM_001137648
	R	TTAACACTGATGGCAAAGAAACAA	
*JAK2*	F	CAGATTTCAGGCCGTCATTT	NM_001030538
	R	ATCCAAGAGCTCCAGTTCGTAT	
*SHP-2*	F	ATGTTGGTGGAGGGGAGAA	NM_204968
	R	GGGGCTGCTTGAGTTGC	
*TAK1*	F	CCAGGAAACGGACAGCAGAG	XM_015284677
	R	GGTTGGTCCCGAGGTAGTGA	
*NFKB1*	F	AGAAAAGCTGGGTCTTGGCA	NM_205134
	R	CCATCTGTGTCAAAGCAGCG	
*ERK1*	F	GCAAGCTTTAGCCCATCCA	NM_204150
	R	GTCATCCAATTCCATATCAAACTT	
*ERK2*	F	CATCGCGACCTCAAACCTTC	AAK56503
	R	TCCGGATCTGCAACACGAG	
*MYD88*	F	GGTTCTGGACAAGACTGGCA	NM_001030962
	R	ATGCTGTAGGAACACCGTGG	
*CSF-1R*	F	ACGGGTAGCCAAGATTTGTG	XM_414597
	R	AGAATGCCGTAGGACCACAC	
*IFN-α*	F	AACCACCCACGACATCCTTC	NM_205427
	R	CAAGCATTGCTCGAGGTGC	
*IFN-β*	F	CTTGCCCACAACAAGACGTG	NM_001024836
	R	GTGTTTTGGAGTGTGTGGGC	
*IFN-γ*	R	AACAACCTTCCTGATGGCGT	NM_205149.1
	F	TGAAGAGTTCATTCGCGGCT	
*IL-17A*	F	TGTCTCCGATCCCTTGTTCT	AM773756
	R	GTCCTGGCCGTATCACCTT	
*IL-17D*	F	ACCCCACAAGATACCCTAAATAC	EF570583
	R	GTGCTGCGGAAGTGAAAAT	
*IL-12p40*	F	AGATGCTGGCAACTACACCTG	NM_213571
	R	CATTTGCCCATTGGAGTCTAC	
*LITAF*	F	AGCTGACGGTGGACCTATTATT	AY765397
	R	GGCTTTGCGCTGGATTC	
*IL-1β*	F	TGCCTGCAGAAG AAGCCTCG	NM_204524
	R	CTCCGCAGCAGTTTGGTCAT	
siCSF1R-1	F	ACAGAUCCCUCAGACACAU	XM_414597
	R	AUGUGUCUGAGGGAUCUGU	
siCSF1R-2	F	GUGAACAGCAAGUUCUACA	XM_414597
	R	UGUAGAACUUGCUGUUCAC	

Restriction enzyme sites are underlined in the forward (*EcoRI*) and reverse primers (*HindIII*). F: forward, R: reverse, No.: number.

## Supplementary Materials

Supplementary materials can be found at http://www.mdpi.com/1422-0067/19/6/1665/s1.

## Abbreviations

ANOVA	analysis of variance
BSA	bovine serum albumin
chIL-34	chicken IL-34
CSF-1R	CSF-1 receptor
CSF-1	colony-stimulating factor 1
DAPI	4′,6-diamidino-2-phenylindole
DMEM	Dulbecco’s modified Eagle’s medium,
ELISA	enzyme-linked immunosorbent assay
ERK1/2	extracellular signal-regulated kinase 1 and 2
FBS	fetal bovine serum
GAPDH	glyceraldehyde-3-phosphate dehydrogenase
HRP	horseradish peroxidase
IL	interleukin
JAK	Janus kinase
MAPK	mitogen-activated protein kinases
M-CSF	macrophage colony-stimulating factor
NF-κB	nuclear factor kappa B
NO	nitric oxide
non-siRNA	nonsense small interfering RNA
p-	phosphorylated
PBS	phosphate-buffered saline
PBST	PBS containing 0.05% TWEEN^®^ 20
PCR	polymerase chain reaction
qRT	quantitative real-time
RT	room temperature
SEM	standard error of the mean
SHP-2	Src homology 2-containing tyrosine phosphatase 2
siRNA	small interfering RNA, siCSF-1R-1
siRNA	sequence that targets intracellular CSF-1R
siCSF-1R-2	siRNA sequence that targets extracellular CSF-1R
SOCS	suppressor of cytokine signaling
STAT	signal transducer and activator of transcription
TAK1	mitogen-activated protein kinase kinase kinase 7
TNF	tumor necrosis factor
TYK2	tyrosine kinase 2
