# Supplementary material for: Interleukin-34 Regulates Th1 and Th17 Cytokine Production by Activating Multiple Signaling Pathways through CSF-1R in Chicken Cell Lines
Source: Int J Mol Sci. 2018 Jun 5;19(6):1665. doi: 10.3390/ijms19061665 (PMC6032434; doi:10.3390/ijms19061665)
Supplement: Supplementary file 1 [file ijms-19-01665-s001.pdf]

Supplementary

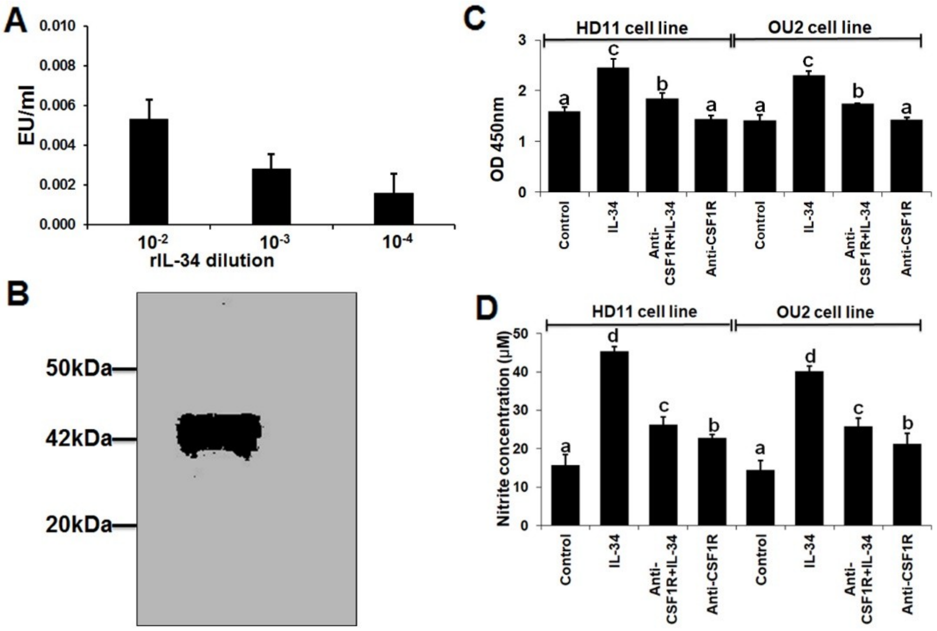

Figure S1. (**A**) Measurement of endotoxin concentrations in the recombinant protein preparations determined by ELISA. A total of 100 μg of protein was used. The initial 100 μg was then diluted 1:100, 1:1000, and 1:10,000 with endotoxin-free PBS. (**B**) Western blot analysis of chIL-34 recombinant protein using anti-His (C-Term)-HRP antibody. The effects of chIL-34 (200 ng/ml), anti-CSF-1R antibody (10μg/ml), and anti-CSF-1R antibody (10μg/ml) and chIL-34 (200 ng/ml) on cell proliferation (**B**) and NO production (**C**) by the HD11 and OU2 cell lines. Data ($n = 3$) are expressed as mean ± SEM of 3 independent experiments. Values with different superscript characters (a, b, c, and d) indicate significant differences between the control and treatment groups as determined by one-way ANOVA ($p < 0.05$).

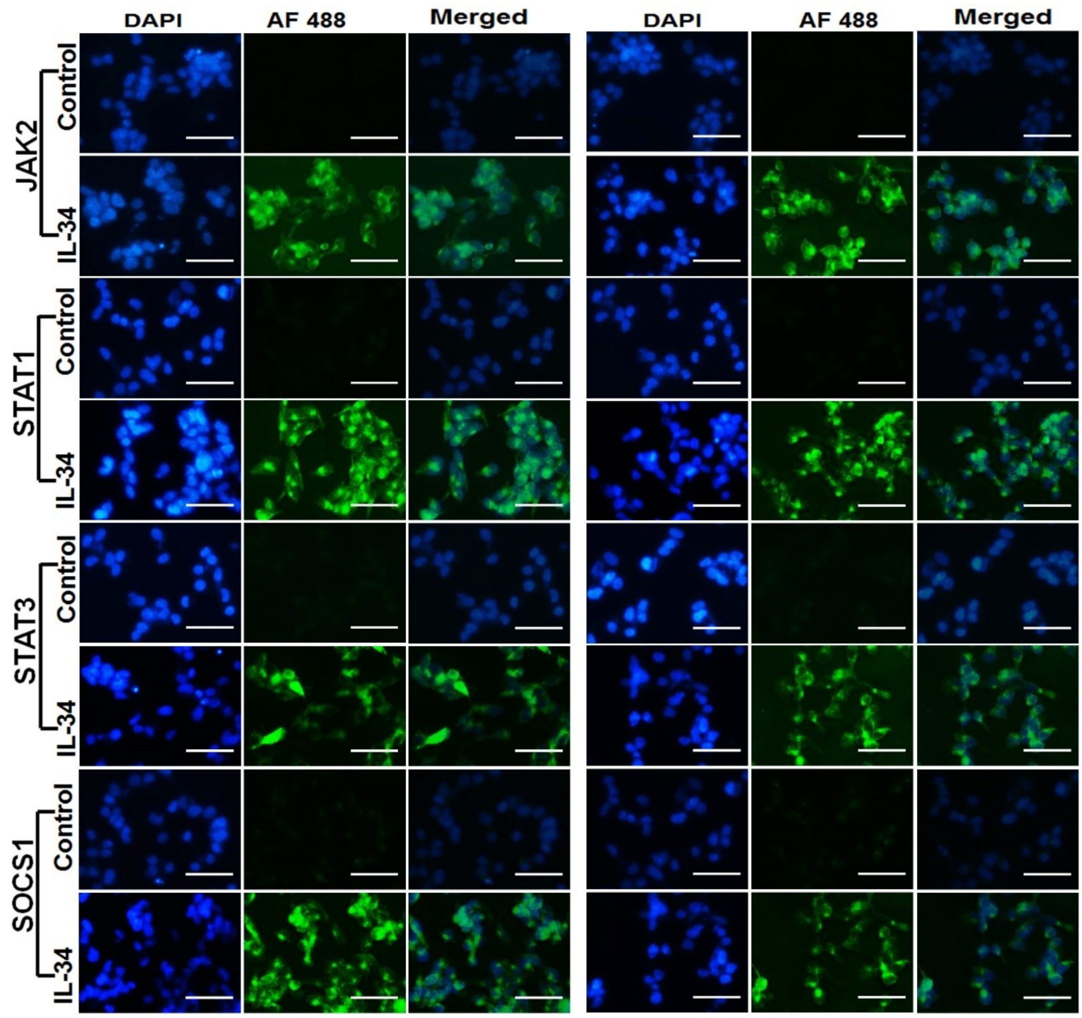

Figure S2. Immunocytochemical analysis of phosphorylated or unphosphorylated JAK/STAT signaling proteins in chicken cell lines. The HD11 (**A**) and OU2 (**B**) cell lines were stimulated with or without chIL-34 (200 ng/ml) for 60 min. Both untreated and treated cells were incubated with primary antibody, Alexa Fluor® 488 goat anti-rabbit IgG (H+L) secondary antibody, and DAPI (blue). Scale bar represents 25 μm.
